# Palliative care for persons with late-stage Alzheimer’s and related dementias and their caregivers: protocol for a randomized clinical trial

**DOI:** 10.1186/s13063-023-07614-4

**Published:** 2023-09-25

**Authors:** M. Toles, C. Kistler, F. C. Lin, M. Lynch, K. Wessell, S. L. Mitchell, L. C. Hanson

**Affiliations:** 1 School of Nursing, University of North Carolina at Chapel Hill, Chapel Hill, NC USA; 2 Department of Family Medicine and Palliative Care Program, School of Medicine, University of North Carolina at Chapel Hill, Chapel Hill, NC USA; 3 Department of Biostatistics, Gillings School of Global Public Health, University of North Carolina at Chapel Hill, Chapel Hill, NC USA; 4 Cecil G. Sheps Center for Health Services Research, University of North Carolina at Chapel Hill, Chapel Hill, NC USA; 5 Hebrew SeniorLife, Hinda and Arthur Marcus Institute for Aging Research, and Department of Medicine, Beth Israel Deaconess Medical Center, Harvard Medical School, Boston, USA; 6 Division of Geriatrics and Palliative Care Program, School of Medicine, University of North Carolina at Chapel Hill, Chapel Hill, NC USA

**Keywords:** Dementia, Randomized and controlled trial, Palliative care, Transitional care

## Abstract

**Background:**

Limited access to specialized palliative care exposes persons with late-stage Alzheimer’s disease and related dementias (ADRD) to burdensome treatment and unnecessary hospitalization and their caregivers to avoidable strain and financial burden. Addressing this unmet need, the purpose of this study was to conduct a randomized clinical trial (RCT) of the ADRD-Palliative Care (ADRD-PC) program.

**Methods:**

The study will use a multisite, RCT design and will be set in five geographically diverse US hospitals. Lead investigators and outcome assessors will be masked. The study will use 1:1 randomization of patient-caregiver dyads, and sites will enroll *N* = 424 dyads of hospitalized patients with late-stage ADRD with their family caregivers. Intervention dyads will receive the ADRD-PC program of (1) dementia-specific palliative care, (2) standardized caregiver education, and (3) transitional care. Control dyads will receive publicly available educational material on dementia caregiving. Outcomes will be measured at 30 days (interim) and 60 days post-discharge. The primary outcome will be 60-day hospital transfers, defined as visits to an emergency department or hospitalization ascertained from health record reviews and caregiver interviews (aim 1). Secondary patient-centered outcomes, ascertained from 30- and 60-day health record reviews and caregiver telephone interviews, will be symptom treatment, symptom control, use of community palliative care or hospice, and new nursing home transitions (aim 2). Secondary caregiver-centered outcomes will be communication about prognosis and goals of care, shared decision-making about hospitalization and other treatments, and caregiver distress (aim 3). Analyses will use intention-to-treat, and pre-specified exploratory analyses will examine the effects of sex as a biologic variable and the GDS stage.

**Discussion:**

The study results will determine the efficacy of an intervention that addresses the extraordinary public health impact of late-stage ADRD and suffering due to symptom distress, burdensome treatments, and caregiver strain. While many caregivers prioritize comfort in late-stage ADRD, shared decision-making is rare. Hospitalization creates an opportunity for dementia-specific palliative care, and the study findings will inform care redesign to advance comprehensive dementia-specific palliative care plus transitional care.

**Trial registration:**

ClinicalTrials.gov NCT04948866. Registered on July 2, 2021.

**Supplementary Information:**

The online version contains supplementary material available at 10.1186/s13063-023-07614-4.

## Introduction

### Background and rationale {6a}

Alzheimer’s disease and related dementias (ADRD) affect 6.5 million Americans and their family caregivers [1]: three million live with late-stage ADRD [2, 3]. ADRD is the only major cause of death with no clinically relevant treatment to prevent, cure, or slow disease progression. ADRD prevalence is expected to double by 2030 [1, 4].

Persons with late-stage ADRD suffer progressive dependency and distressing symptoms. Late-stage ADRD, defined as Global Deterioration Scale (GDS) 5–7, is characterized by moderately severe to very severe cognitive impairment and the need for daily caregiver support for activities of daily living (ADLs) [5]. Though life expectancy may be months to years, distressing symptoms of ADRD and co-morbid conditions are prevalent [6–8]. Neuropsychiatric symptoms—such as physical or verbal aggression, socially inappropriate behaviors, and sleep–wake cycle disruptions—occur in late-stage dementia and cause suffering for the person with ADRD and strain for caregivers [9, 10].

Caregivers of persons with late-stage ADRD experience physical, emotional, and financial strain [11–13]. Care in late-stage ADRD is demanding and requires substantial formal and informal resources. While most persons with ADRD at Global Deterioration Scale (GDS) stage 7 ADRD live in nursing homes, a majority of persons at GDS 5–6 live at home [3]. Moreover, persons with late-stage ADRD experience frequent transitions between home, long-term care facilities, and hospitals; thus, caregivers confront challenges coordinating care between settings and providers of care [14–16].

Persons with late-stage ADRD are frequently hospitalized [15, 16], and acute illness, such as pneumonia, other infections, and falls, exacerbates pain and neuropsychiatric symptom distress and signals worse prognosis [17–19]. Moreover, hospitalization may trigger burdensome treatments [20, 21]. Hospitalization is not always medically necessary or beneficial; research indicates that 23–47% of ADRD hospitalizations are potentially avoidable [22, 23]. During hospitalization, caregivers face complex decision-making obligations. Shared decision-making and access to out-of-hospital treatment are essential to avoid unwanted hospitalizations [17–19, 22].

In prior research, caregivers of persons with late-stage ADRD prioritized the goal of comfort over life prolongation [24, 25]. For example, a majority of caregivers in the Goals of Care clinical trial prioritized the goal of comfort over function or life prolongation: 57% for GDS 5, 62% for GDS 6, and 84% GDS 7 [19]. While ADRD caregivers face complex and morally distressing choices as surrogate decision-makers [14, 26], many report poor quality communication and gaps in shared decision-making for late-stage ADRD [23, 27–29]. Only 17% of nursing home residents have a living will, and only 38% of family decision-makers for persons with GDS 7 ADRD recall involvement in major medical decisions [25, 30, 31].

Acute illness is a sentinel event in ADRD, signaling an important opportunity to access palliative care teams, which are present in 72% of US hospitals [32–34]. Comprehensive palliative care includes (1) counseling for prognostic awareness, (2) symptom management, (3) shared decision-making to align treatment with goals, and (4) enhanced support for emotional, spiritual, and practical needs [35]. Interdisciplinary teams of physicians, advance practice providers, nurses, social workers, and chaplains provide palliative care. For patients with cancer and other serious illnesses and their caregivers, palliative care improves the quality of life, symptoms, and care [36, 37]; it reduces treatment intensity without affecting survival [38–41], and unlike hospice, palliative care is appropriate even when life prolongation remains the goal. However, persons with ADRD and their caregivers have unique palliative care needs that do not match standard models of palliative care.

**Fig. 1:**
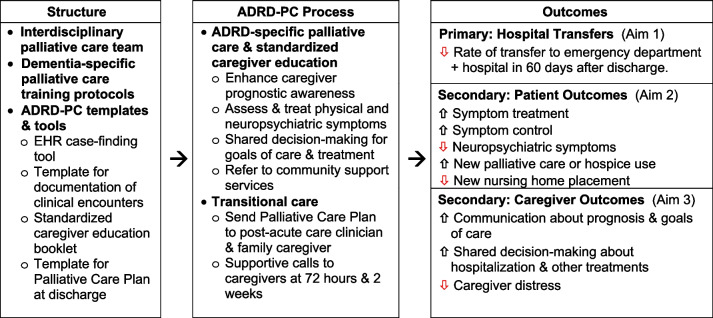
Alzheimer’s disease and related dementias-palliative care (ADRD-PC) intervention model [46]

Building on Alzheimer’s Association Practice Recommendations [42], the Advanced Dementia Consult Service study [43], and other evidence for the unique palliative care needs in ADRD [44, 45], the study team designed the Alzheimer’s Disease and Related Dementias-Palliative Care (ADRD-PC) intervention. As described in Fig. 1 and below, the ADRD-PC intervention model includes (1) structural elements of healthcare (e.g., EHR case-finding tool) and (2) care processes (i.e., dementia-specific palliative care, standardized caregiver education, and transitional care) that are designed to improve (3) patient and caregiver outcomes, such as hospital transfers, symptoms, and caregiver distress [46, 47]. In the randomized controlled ADRD-PC pilot study, 62 dyads of hospitalized patients with GDS 5–7 ADRD and their family caregivers were randomized to ADRD-PC vs usual care with publicly available educational material [48]. While intentionally not designed with adequate power to show a difference in 60-day hospital transfers (rate ratio 1.28 (95% CI 0.52, 3.22)), findings indicated that intervention dyads were more likely to have an active Physician Orders for Life-Sustaining Treatment (POLST) [49] advance directive (79% vs 30%, *p* < 0.001) and to make a decision to avoid future hospitalization (13% vs 0%, *p* = 0.033) [48]. With ADRD-PC, persons with late-stage ADRD had more palliative care domains addressed in their treatment plan (Palliative Care Domain Index Score 7.6 vs 2.7, *p* < 0.001, range 0–10), with increased symptom management for dyspnea (77% vs 34%, *p* < 0.001), constipation (93% vs 25%, *p* < 0.001), depression (83% vs 25%, *p* < 0.001), and delirium (80% vs 19%, *p* < 0.001) [48]. Moreover, caregivers were more likely to discuss prognosis (90% vs 3%, *p* < 0.001) and goals of care (90% vs 25%, *p* < 0.001) [48].

## Objectives {7}

Our research objective is to conduct a multi-site efficacy randomized clinical trial (RCT) of the ADRD-PC program. We will enroll 424 dyads of hospitalized patients with late-stage ADRD (GDS 6–7 or GDS 5 with significant co-morbidity) with their family caregivers at 5 geographically diverse sites in the USA. Our primary hypothesis is that ADRD-PC will reduce hospital transfers (aim 1). The specific aims are as follows:

- Aim 1: To conduct a multi-site RCT of the ADRD-PC program (intervention arm) vs publicly available educational material for dementia caregivers (control arm) to compare 60-day hospital transfers (hospitalization and emergency room visits) for persons with late-stage ADRD (primary outcome). H1: 60-day hospital transfers will be lower in the intervention vs control arm.
- Aim 2: To compare patient-centered secondary outcomes between the intervention and control arms: (a) symptom treatment (Palliative Care Domain Index [23]), (b) symptom control (Symptom Management at the End of Life in Dementia Scale [50] and Neuropsychiatric Inventory Questionnaire [51]), (c) post-acute use of community palliative care or hospice, and (d) new nursing home transitions. Patients in the intervention vs control arms will have better symptom treatment (H2a) and symptom control (H2b), increased use of community palliative care or hospice (H2c), and fewer new nursing home transitions at 60 days post-discharge (H2d).
- Aim 3: To compare caregiver-centered secondary outcomes between the intervention and control arms: (a) communication about prognosis and goals of care, (b) shared decision-making, and (c) caregiver distress (Family Distress in Advanced Dementia Scale [52]). Caregivers in the intervention vs control arms will more often have documented communication about prognosis and goals of care (H3a) and report more shared decision-making about hospitalization and treatments (H3b) and less caregiver distress at 60 days post-discharge (H3c).

## Trial design {8}

The design is an RCT conforming to SPIRIT and CONSORT statements for trial methods and protocol [53, 54]. The approach uses NIA standards for a multi-site efficacy trial (stage II) of a protocolized behavioral intervention, while incorporating pragmatic features to support sustainability and future dissemination [55].

## Methods: participants, interventions, and outcomes

### Study setting {9}

The study setting will be five US medical centers with interdisciplinary palliative care teams: University of North Carolina at Chapel Hill Medical Center (UNC), Massachusetts General Hospital/Harvard Medical School (MGH), Eskenazi Health affiliated with Indiana University (IU), UCHealth University of Colorado Hospital (UC), and Emory University Healthcare System (EU). Study sites are members of the Palliative Care Research Cooperative (PCRC) and utilize the Epic electronic health record system. Sites were recruited by a standardized PCRC process that seeks clinical trial sites with study-specific clinical practice resources matched to trial recruitment needs.

### Site preparation

Before enrollment of study participants, the principal investigator (PI) and project manager will provide consultation for site-based teams to incorporate ADRD-PC tools in hospital electronic health records (EHR) systems. New tools will include the screening/case finding algorithm, the template for documenting clinical encounters, and the template for the Palliative Care Plan at discharge. The PI, project manager, and clinical research coordinator (CRC) will provide 4 h of research protocol training for site clinical research coordinators (site-CRCs) and site principal investigators (site-PIs) [56]. The project manager and CRC will also provide 4 h of training for the site-CRCs. Training content will include implementation of EHR screening, procedures for recruitment and enrollment, interview and EHR review data collection, detection and adverse event data capture, and regulatory procedures [48]. During this same time period, the PI will provide 4 h of training for the members of the interdisciplinary palliative care teams in each study hospital who will provide clinical consultation as part of the ADRD-PC program, focused on four domains: (1) prognostic awareness, (2) physical and neuropsychiatric symptoms, (3) shared decision-making for goals of care and key treatment choices in ADRD, and (4) transitional care to enhance post-acute support.

### Eligibility criteria {10}

The study will enroll 424 dyads of hospitalized persons with late-stage ADRD and their family caregivers; dyads will participate for 60 days following the index hospitalization. The eligibility criteria for persons with ADRD are (1) aged 55 or older, (2) hospitalized, (3) have a physician-confirmed diagnosis of ADRD, and (4) staged GDS 6 or 7 or GDS 5 with additional co-morbidity defined by Charlson Comorbidity Index scored > 5. As in the pilot, the ADRD stage will be confirmed by the attending physician and caregiver. The eligibility criteria for caregivers are (1) the adult (aged 18 or older) legally authorized representative (LAR) for healthcare and has the capacity to serve in this role, (2) support the person with ADRD, and (3) can complete interviews in English. As in our prior ADRD research, other family caregivers may be present per primary caregiver request, but only the primary caregiver is a participant. Dyads will be excluded if (1) the LAR is not a family caregiver, (2) the patient currently receives palliative care or hospice, or (3) patient or caregiver would be unduly stressed.

### Recruitment {15}

#### Participant screening procedures

To recruit study participants, the site-CRCs will use rapid case-finding methods from the ADRD-PC pilot RCT, reviewing the lists generated by EHR screening algorithms to identify persons aged 55 and older with ADRD within 24 h of hospitalization [48]. After a brief EHR review to confirm other eligibility criteria including evidence for ADRD stage and Charlson Comorbidity Index scoring, site-PIs will contact attending physicians and seek permission for the site-CRCs to approach caregivers of the person with ADRD. Attending physicians, who are in direct contact with the person with ADRD and their caregiver, confirm ADRD staging.

#### Participant recruitment and enrollment procedures

Family caregivers may be present in the hospital, barred from visitation due to COVID-19 restrictions and safety concerns, or may live at a great distance from the hospital; thus, recruitment and enrollment procedures will include both in-person and telephone/virtual communication methods.

Site-CRCs will introduce the study to eligible family caregivers using scripted telephone calls or in-person visits. When the site-CRC conducts recruitment and enrollment via telephone, he or she will ask if the individual is in a private location and feels comfortable talking about the study at that time. The site-CRC will schedule an alternative time if necessary.

#### Informed consent {26a}

The family caregiver will provide informed consent for themselves and for all persons with ADRD as they lack decisional capacity. If the family caregiver chooses to participate, the site-CRC will conduct the informed consent interview in person or over the phone. If the family caregiver agrees to participate, study informational materials and the informed consent form will be sent to them by mail or secure e-mail. Due to the low risk of study participation and the restrictions on immediate written consent described above, verbal consent will be accepted for participation in the enrollment caregiver interview and for randomization with initiation of the ADRD-PC or control conditions.

#### Additional consent provisions for collection and use of participant data and biological specimens {26b}

Not applicable as no additional consent provisions for ancillary studies are included.

### Assignment of interventions: allocation

#### Sequence generation {16a}

Randomization will occur following the enrollment interview, with the dyad as the unit of randomization and analysis. To ensure concealed allocation of randomization, the study statistician will create site-specific, computer-generated random treatment assignments to assign dyads in a 1:1 ratio to intervention or control arms.

#### Concealment mechanism {16b}

The randomization will be generated by the study statistician within the REDCap software and will remain concealed until the evaluation of the study aims [57].

#### Implementation {16c}

After the allocation sequence is generated, each patient/caregiver dyad will be assigned a study ID number in each site. The site-CRC will reveal allocation to the site-PIs, who will communicate study arm assignment to the attending physician and to the palliative care team to initiate ADRD-PC consultation (intervention), or site-CRCs will deliver a copy of publicly available dementia caregiver educational material (control).

### Interventions

#### Intervention description {11a}

Patient-caregiver dyads that are randomized to the intervention will receive the ADRD-PC program that includes speciality palliative care consultation during hospitalization and telephone-based transitional care over 2 weeks after discharge. ADRD-PC dementia-specific palliative care, standardized caregiver education, and transitional care are described below.

#### Dementia-specific palliative care

Members of the site-based, interdisciplinary palliative care team, including a physician or advanced practice provider and at least one additional discipline (nurse, social worker, chaplain), will visit the dyad in the hospital until hospital discharge. Palliative care clinicians will address four domains: (1) prognosis—exploration and communication of stage, trajectory, and prognostic awareness; (2) symptoms—assessment and treatment for physical and neuropsychiatric symptoms; (3) shared decision-making—exploration of values and goals of care and discussion of treatment decisions; and (4) transitional care—assessment of needs, care planning, and recommendation for support services. Discussions may include exploration of the overall goals of care and decision-making about potentially burdensome treatments of resuscitation, ventilator use, feeding tubes, antibiotics, or future hospitalizations. When clinically appropriate, consultation will also include the completion of new advance directives, such as a Physician Orders for Life-Sustaining Treatment (POLST) [49], or comparable portable order set to document decisions for current care plans. Palliative care clinicians will document care in the EHR with the ADRD-PC Note Template.

#### Standardized caregiver education

Members of the site-based, interdisciplinary palliative care team will share and discuss the booklet, “Advanced Dementia: A Guide for Families,” which addresses common concerns and treatment decisions [45]. In discussion with the dyad, clinicians will individualize the content of the booklet with counseling about topics including (1) dementia and its stages; (2) determining the primary goal of care; (3) approaches to decision-making, eating problems, decisions about hospitalization, and decisions for infections; (4) discussing how dementia affects the family; and (5) explaining differences between hospice and palliative care.

#### Transitional care

Members of the site-based, interdisciplinary palliative care team will also provide transitional care at three time points [58]. Pre-discharge, a member of the palliative care team will create an individualized, templated palliative care plan and provide copies to the primary post-acute clinician and the family caregiver. This document will summarize recommendations in the four domains and provide contact information for the palliative care team for follow-up questions. Near discharge, the palliative care team will explore the adequacy of patient and caregiver support, such as community palliative care, hospice, and Alzheimer’s disease caregiver support groups. Post-discharge, a designated palliative care team member (usually a social worker or case manager) will call the family caregiver within 72 h and again 2 weeks after discharge. The purpose of these calls will be to support the implementation of the palliative care plan and promote access to post-acute services.

#### Control condition

Patient-family caregiver dyads randomized to the control arm will receive educational materials from the Alzheimer’s Association, specifically designed for late-stage ADRD caregivers, delivered by the site-CRCs [59]. The patient will receive usual hospital and post-acute care.

#### Strategies to improve adherence to interventions {11c}

As described in Table 1, the NIH Behavior Change Consortium standards will be used to evaluate the degree to which clinicians use the ADRD-PC protocol as intended [60].

**Table 1:** Strategies to improve adherence to interventions [60]

Type of fidelity	Procedures to ensure fidelity	Fidelity monitoring
Study design	• Intervention based on a well-defined protocol • Standardized tools and templates	• Protocol review and version control supported by the Palliative Care Research Cooperative Group
Standardized training and delivery	• Training of Site-PIs on study protocol and SOPs • Training of site CRCs on study protocol and SOPs • Training of PC clinicians to deliver ADRD-PC • Audio-recorded training modules for consistent re-training or for new personnel • ADRD tools and templates	• Training material review by the Palliative Care Research Cooperative Group • Completed training • Post-training evaluation for PC clinicians (threshold score 80%) • Delivery of tools and templates to all study sites
ADRD-PC intervention enactment	• Monthly conference calls (led by PI) with site PC clinicians • Monthly conference calls (led by UNC project manager) with site CRCs and site PIs • Utilization of ADRD-PC Program with standardized content areas • Documentation of clinical encounters in site medical record	• Site-CRC tracking of completion of 4 ADRD-PC components: PC encounter, caregiver education, delivery of PC plan to caregiver and primary or post-acute care provider, and completion of 2 transitional care calls • Threshold score 80% of dyads with completion of 4 ADRD-PC components • Site-specific feedback when fidelity drops below the threshold • Review 10% random of deidentified ADRD-PC encounter notes to ensure adherence, scored for quality of content across 4 clinical domains

*SOP* Standard operating procedures, *Site-PI* Site principal investigator, *PC* Palliative care, *ADRD-PC* Alzheimer’s Disease and Related Dementias-Palliative Care Intervention, *CRC* Clinical research coordinator

The PI will review monthly tracking reports of fidelity metrics and will use teleconferences to provide monthly coaching calls to site-CRCs, site-PIs, and clinicians. Coaching will be tailored and may include a review of study procedures, feedback on enrollment, retention and fidelity data, and strategies to overcome barriers. Sites failing to meet fidelity will be given site-specific feedback and re-training.

### Assignment of interventions: blinding

#### Who will be blinded {17a}

The lead investigator, a co-investigator (MT), a study consultant (SM), and outcome assessors will be masked. They will remain masked to the study arm assignments until analysis; however, site-PIs, including one study co-investigator (CK), and the study statistician (FCL) will not be masked due to their roles in communicating study arm assignments and adverse event reporting. Site-CRCs will be masked to the study arm to collect outcome measures in family caregiver interviews. Since the EHR in study sites will have data regarding ADRD-PC palliative care encounters, site-CRCs will complete follow-up EHR reviews after 60-day follow-up interviews are completed and study participation ends for each dyad.

#### Procedure for unblinding if needed {17b}

If an unanticipated serious adverse event occurs, the IRB and the DSMB will consider unblinding prior to the final analyses if judged necessary to address participant safety.

### Outcomes {12}

The description of study outcomes, measures and method of aggregation, data sources, and timing of data collection are reported in Table 2 and the narrative that follows. The primary outcome, 60-day hospital transfers, will be measured by the number of emergency room visits plus hospital admissions within 60 days after discharge from the index hospitalization. CRCs will collect this outcome data using 60-day EHR reviews and 30- and 60-day follow-up telephone interviews with family caregivers (Table 2). The 60-day hospital transfer outcome was selected owing to its importance to patients and caregivers and as a marker of healthcare cost [15, 61].

**Table 2:** Primary and secondary outcomes

Outcome	Measure and method of aggregation	Source	Timing
Aim 1: Primary outcome			
Hospital transfers	Hospital transfer count—number of emergency room visits + hospital admissions within 60 days after discharge from index hospitalization. Method of aggregation—the count of hospital transfers in intervention and control groups	Caregiver/EHR review	30 and 60 days after discharge
Aim 2: Secondary patient outcomes			
Symptom treatment	Palliative Care Domain Index items—ten domains of palliative care are scored as present or absent (range 0–10 with higher scores indicating increased symptom treatment) [23]. Method of aggregation—the mean score in intervention and control groups	EHR review	60 days after discharge
Symptom control (physical and neuropsychiatric symptoms)	Symptom management at the end of life in dementia—0–5 Likert-scaled measure of 9 symptoms during 30-day look-back. The range is 0–45 with higher scores indicating better symptom control [50]. Method of aggregation—the mean score in intervention and control groups Neuropsychiatric Inventory Questionnaire—presence, frequency, and severity of 12 neuropsychiatric symptoms. The range is 0–36 and 0–60 with higher scores indicating worse symptom control [51]. Method of aggregation—the mean scores in intervention and control groups	Caregiver	30 and 60 days after discharge
Use of hospice or community palliative care	Rate of utilization—% of persons with ADRD who access hospice or community palliative care services from discharge. The count of hospital use of intervention and control groups. Method of aggregation—the proportion of patients who do and do not use post-acute palliative care or hospice in intervention and control groups	Caregiver/EHR review	30 and 60 days after discharge
Transition to nursing home	Rate of transition—% of persons with ADRD who transition to nursing home care. Method of aggregation—the proportion of patients who do and do not transition to nursing home care in intervention and control groups	Caregiver/EHR review	60 days after discharge
Aim 3: Secondary caregiver outcomes			
Documented discussion of dementia prognosis	Rate (%) of caregivers with a documented discussion of dementia prognosis during the index hospitalization. Documentation must include evidence of information sharing between a clinician and family caregiver regarding dementia stage or trajectory, life expectancy, or future function. Method of aggregation—the proportion of caregivers who do and do not participate in a discussion about dementia prognosis in intervention and control groups	EHR review	60 days after discharge
Documented discussion of goals of care	Rate (%) of caregivers with a documented discussion of overall goals of care during the index hospitalization. Documentation must include evidence of shared decision-making between a clinician and family caregiver about a choice to guide overall treatment using comfort, function, survival, or other stated goals of healthcare. Method of aggregation—the proportion of caregivers who do and do not participate in communication about goals of care in intervention and control groups	EHR review	60 days after discharge
Shared decision-making—about hospitalization	Rate (%) of caregivers reporting shared decision-making about a future hospitalization decision. Measured with a single item asking if caregivers made a decision in discussion with a treating clinician to accept or avoid future hospitalizations. Method of aggregation—the proportion of caregivers who do and do not participate in shared decision-making about hospitalization in intervention and control groups	Caregiver	30 and 60 days after discharge
Shared decision-making—about burdensome treatments	Rate (%) of caregivers reporting shared decision-making about resuscitation, ventilator use, tube feeding, and antibiotics for infection treatment. Measured with items asking if caregivers made a decision in discussion with a treating clinician about resuscitation, ventilator use, tube feeding, and antibiotics for infection treatment. Method of aggregation—the proportion of caregivers who do and do not participate in shared decision-making about burdensome treatments in intervention and control groups	Caregiver	30 and 60 days after discharge
Caregiver distress	Family Distress in Advanced Dementia Scale—21 Likert-scaled items measured 3 domains of distress—emotional, dementia preparedness, and experience of care. The range is 1–5 with higher scores indicating more distress [52]. Method of aggregation—the mean score in intervention and control groups	Caregiver	Enrollment and 30 and 60 days after discharge
Caregiver burden	Zarit Burden Scale, short form. The range 0–24 with higher scores indicating more burden [62]. Method of aggregation—the mean score in intervention and control groups	Caregiver	Enrollment and 30 and 60 days after discharge

*ADRD* Alzheimer’s disease and related dementias

Secondary patient outcomes will include (1) symptom treatment as measured by Palliative Care Domain Index items [23], which has 10 items scored present vs absent, ranging from 0 to 10 with higher scores indicating increased symptom treatment; (2) symptom control for physical symptoms as measured by the Symptom Management at the End of Life in Dementia (SM-EOLD) [50], scored from 0 to 45 with higher scores indicating better symptom control; (3) symptom control for neuropsychiatric symptoms as measured by the Neuropsychiatric Inventory Questionnaire (NPI-Q) [51], which has a range in two subscales of 0–36 and 0–60, with higher scores indicating worse symptom control; (4) access to hospice as measured by the percentage of people with ADRD who access hospice services; (5) access to community-based palliative care as measured by the percentage of people with ADRD who access community-based palliative care services; and (6) transition to nursing home level of care as measured by the percentage of people with ADRD who transition to nursing home care. As described in Table 2, CRCs will collect secondary patient outcomes data in 60-day EHR reviews and/or follow-up telephone interviews with family caregivers 30 and 60 days after discharge from the index hospitalization.

Secondary caregiver outcomes will include (1) documented discussion of dementia prognosis as measured by the percentage of caregivers with documented discussion of dementia prognosis during the index hospitalization; (2) documented discussion of goals of care as measured by the percentage of caregivers with documented discussion of goals of care during the index hospitalization; (3) shared decision-making regarding hospitalization as measured by the percentage of caregivers reporting shared decision-making about future hospitalization; (4) shared decision-making regarding burdensome treatment as measured by the percentage of caregivers reporting shared decision-making about resuscitation, ventilator use, tube feeding, and antibiotics for infection treatment; (5) caregiver distress as measured by the Family Distress in Advanced Dementia Scale [52], which ranges from 1 to 5 with higher scores indicating more distress; and (6) caregiver burden as measured by the Zarit Burden Scale short form [62], which ranges from 0 to 24 with higher scores indicating more burden. As described in Table 2, CRCs will collect secondary caregiver outcomes data in 60-day EHR reviews and/or follow-up telephone interviews with family caregivers 30 and 60 days after discharge from the index hospitalization.

In addition to these outcomes, data on covariates will be collected through EHR review and interviews with family caregivers. Patient demographics will include age, sex, race and ethnicity, and marital status. The pre-admission residence will be measured as a private home, assisted living facility, nursing home, or others. Patient co-morbidity will be measured using the Charlson Comorbidity Index, a validated, widely used index with weighted scores for age and diagnoses [63]. Functional status will be measured using the Bedford Alzheimer Nursing Severity (BANS) Scale for function in late-stage dementia [64]. This scale ranges from 0 to 28, with higher scores indicating worse function (alpha = 0.80, Pearson’s *r* 0.62–0.79) [64]. ADRD stage will be measured using the GDS, and etiology will be defined by an attending physician during recruitment and enrollment [5]. Advance directives will be measured as the presence or absence of a living will, Health Care Power of Attorney, portable DNR order, or POLST order set form [49]. Survival will be measured for each patient as days from study enrollment to death up to 60 days follow-up. Caregiver demographics will include health condition, age, sex, race and ethnicity, relationship to the patient, self-report of health status (excellent, very good, good, fair, poor), depression (PHQ-2), marital status, education level, current work situation, and social determinants of health [65]. Family caregiver perception of prognosis will be measured using a single item asking what the caregiver expects will happen to the patient during the next 6 months, with response options of “get better,” “stay about the same,” “get worse,” or “likely to die.”

#### Participant timeline {13}

Participant flow is shown in Table 3.

**Table 3:** Schedule of enrollment, interventions, and assessments

	Visit 1: During hospitalization	Visit 2: During hospitalization (intervention)	Visit 3: Palliative care call 72 h after discharge (intervention)	Visit 4: Palliative care call 2 weeks after discharge (intervention)	Visit 5 CRC interview with caregiver in 30 days after discharge	Visit 6 CRC interview with caregiver in 60 days after discharge	EHR review post-60-day hospitalization
**Study enrollment activities**							
EHR eligibility screen	X						
Informed consent	X						
Enrollment baseline assessment	X						
Randomization	X						
Control arm	X				X	X	X
ADRD-PC intervention arm	X	X	X	X	X	X	X
**Data collection activities**							
Hospital/emergency department transfers					X	X	X
Palliative Care Domain Index							X
Symptom management at the end of life in dementia					X	X	
Neuropsychiatric Inventory Questionnaire					X	X	
Post-acute use of palliative care, hospice					X	X	
New nursing home transition							X
Documented discussion of dementia prognosis							X
Documented decision-making for goals of care							X
Shared decision-making about hospitalization and burdensome treatments					X	X	
Family Distress in Advanced Dementia Scale					X	X	
Zarit Burden Scale					X	X	

*ADRD-PC* Alzheimer’s Disease and Related Dementias-Palliative Care, *CRC* Clinical research coordinator, *EHR* Electronic health record

#### Sample size {14}

Sites will enroll *N* = 424 dyads of hospitalized patients with late-stage ADRD (GDS 6–7 or GDS 5 with significant co-morbidity) with their family caregivers. Statistical power is based on a 2-sided alpha of 0.05 significant tests and using standard deviation estimates from our pilot study (Table 4). Power calculations assume a “best guess” 15% dropout (death, withdrawal) rate based on prior ADRD research (*n* = 180 per group at 60-day follow-up) and a “worst case” 25% dropout rate (*n* = 159 per group). These rates consider 9% patient mortality and 92% caregiver retention rate in the pilot. In our pilot data, 0.52 is the lower bound of the 95% confidence interval for the incidence rate ratio (IRR) comparing intervention vs control for 60-day hospital transfers. This corresponds to an average absolute reduction of 0.26 transfers per 60-day period due to intervention, assuming the pilot study baseline control rate of 0.53. These power calculations consider reductions of 0.25 and 0.20 transfers, which are plausible values within the confidence bounds in the ADRD-PC pilot study [48]. In Table 4, an “optimistic” reference power calculation is given assuming a Poisson model (i.e., variance equals the mean), and a second “realistic” calculation assumes overdispersion. In the latter case, the standard deviation of the number of transfers is assumed to be 30% greater than that in the Poisson model based on an estimated variance inflation factor in our pilot data of 1.69 (i.e.,√1.69 = 1.3). Allowing for overdispersion, we expect 81% power to detect a reduction in 60-day hospital transfers of 0.25, assuming a 15% dropout. For the number of palliative care domains for aim 2 (to cite one example of a secondary outcome), we expect more than 99% power to detect at least a 5-point improvement in the intervention group vs the control group at 60 days.

**Table 4:** Power for comparing intervention and control arms based on the study design

Aim	Measure	Control	SD	Difference	Power, 15% dropout	Power, 25% dropout
**1**	60-day hospital transfers (Poisson rate)	0.53	–	0.25, 0.2	0.96, 0.82	0.95, 0.79
	60-day hospital transfers (over-dispersed)	0.53	–	0.25, 0.2	0.81, 0.60	0.76, 0.55
**2**	PC Domain Index (0–10)	2.7	1.7	5, 0.5	> 0.99, 0.80	> 0.99, 0.75
	60-day hospice use (%)	3%	–	22%, 8%	> 0.99, 0.85	> 0.99,0.80
	Symptom distress (SM-EOLD)	36.4	7.8	4, 2.3	> 0.99, 0.80	> 0.99, 0.75
	Transition to nursing home care (%)	33%	–	–	> 0.99, 0.82	> 0.99, 0.78
**3**	Discussion of dementia prognosis	3%	–	–	> 0.99, 0.85	> 0.99, 0.80
	Discussion of goals of care (%)	25%	–	–	> 0.99, 0.82	> 0.99, 0.77
	Decision-making about hospitalization	0%	–	–	> 0.99, 0.78	> 0.99, 0.73
	Decision-making about treatments	6%	–	47%, 9%	> 0.99, 0.80	> 0.99, 0.75
	Family distress in advanced dementia	2.4	0.5	0.15, 0.1	0.81, 0.47	0.76, 0.43

*SD* Standard deviation, *PC* Palliative care, *SM-EOLD* Symptom management at the end of life in dementia

### Data collection and management

#### Plans for assessment and collection of outcomes {18a}

After informed consent is obtained, data collection will be identical for both arms. Data will be obtained from caregiver enrollment interviews and 30- and 60-day interviews post-discharge and from electronic health record (EHR) reviews encompassing the 60-day period post-hospitalization. Based on the pilot RCT, we estimate 10% of persons with late-stage ADRD will die during follow-up; in these cases, caregiver interview data collection will proceed using a Bereavement Interview adaptation. Outcome measures will focus on 60-day interviews; 30-day interim interviews are necessary to support retention, ensure data capture for persons with ADRD who die, and for valid recall of hospital transfers (primary outcome) and secondary outcomes.

#### Plans to promote participant retention and complete follow-up {18b}

Retention is supported by transitional care calls (on days 3 and 14 after discharge), appointments for follow-up interviews with recognizable CRC names and phone numbers, and a $25 gift card per interview.

#### Data management {19}

Data collection will be the responsibility of the site-based CRCs under the supervision of the site-based PI, with overall supervision from the PI and the project manager; the site-PIs will be responsible for ensuring the accuracy, completeness, legibility, and timeliness of the data reported.

All research data will be entered into REDCap, a 21 CFR Part 11-compliant data capture system provided by UNC [57]. REDCap includes password protection and internal quality checks, such as automatic range checks, to identify data that appear inconsistent, incomplete, or inaccurate. Clinical data will be entered directly from the source documents [57].

#### Confidentiality {27}

Participant confidentiality and privacy will strictly be held in trust by the participating investigators, their staff, the safety and oversight monitor(s), and the sponsor(s) and funding agency. This confidentiality is extended to the data being collected as part of this study. No personally identifiable information from the study will be released to any unauthorized third party without prior written approval of the sponsor/funding agency. The study participant’s contact information will be securely stored at each clinical site for internal use during the study. Data will be entered into a password-protected, secure database, and all paper documentation will be maintained in locked files. The Sheps Information Technology group, at UNC, enables standard operating procedures required to secure the network and databases, including operational and technical controls. All servers are located within a hardened data center.

## Statistical methods

### Statistical methods for primary and secondary outcomes {20a}

Patient and caregiver dyads will be the primary unity of analysis. Descriptive analyses of variables will examine distributions, influential data points, and missing data. All a priori analyses, primary and subgroup, will use intention-to-treat analysis. The effectiveness of randomization will be evaluated by comparing intervention and control participants on baseline measures because variables that are not equally distributed between arms could potentially bias results. We will include these variables in each initial model and use a change-in-effect method for determining whether they are confounders. Given our randomized design, we anticipate little confounding. A priori exploratory analyses will examine the effects by sex, race/ethnicity, and GDS stage.

#### Aim 1 analysis

The control and intervention arms will be compared on hospital transfers (primary outcome) at 60 days post-discharge. This count outcome is defined as the total number of emergency room visits plus hospital admissions (including observation stays) per at-risk patient-days. Poisson regression with empirical “robust” standard errors allowing for overdispersion will be used to compare the transfer rates between groups. Length of follow-up will be used as the offset variable, which means that patients who have data only from the 30-day interview will be included. The primary analysis will be based on the model with the main effects for the treatment group, study site, GDS stage, and patient and caregiver sex as biological variables. Evaluation of the treatment effect will be based on a Wald test. The covariate-adjusted treatment effect will be quantified as an IRR with a 95% confidence interval. Considering that over 60% of patients in the pilot study did not have any hospital transfers, a sensitivity analysis will be conducted for the treatment effect on 60-day hospital transfers with a marginalized zero-inflated Poisson (MZIP) model [66], which may modestly increase power. Secondary Poisson and MZIP analyses will compare study arms for 30-day hospital transfer rates.

An exploratory analysis will be conducted to examine the interactions of the treatment group with study site, sex, race/ethnicity, and GDS Stage on 60-day hospital transfers [5]. An interaction model will be used if an omnibus test for interactions is statistically significant at the 0.05 level. A second exploratory analysis will evaluate a dose–response effect of the intervention on the hospital transfer rate. A dose for patients receiving the intervention is defined as the number of key intervention components used in fidelity monitoring (range 0 to 4 with 0 for controls). The dose–effect is the incremental benefit of adding a single component; its rescaling gives the per-protocol effect of the full intervention.

#### Aim 2 analysis

As described below, the control and intervention arms will be compared on patient secondary outcomes at 60 days post-discharge on the following outcomes:

- Symptom treatment: This ordinal outcome is the summation of presence (= 1) or absence (= 0) of 10 domains of palliative care [23]. It will be treated as a continuous variable due to a high variation observed in the pilot data. Student’s *t*-test will be used to compare the mean difference between the study arms unless violation of assumptions warrants the Mann–Whitney test. A multiple linear regression model will be used with main effects for treatment group, study site, GDS stage, and patient and caregiver sex as a biological variable.
- Symptom control: The linear mixed models, with random intercepts for caregiver report of patients’ symptom distress (SM-EOLD) [50] and caregiver report of patients’ neuropsychiatric symptom distress (NPI-Q) [51], will be used with a time indicator (30 vs 60 days follow-up) with the above main effects.
- Use of post-acute palliative care or hospice: This outcome will be defined as whether patients ever use hospice or outpatient palliative care during the time from discharge to 60 days follow-up. The proportions of those who do and do not use post-acute palliative care or hospice will be compared using Pearson’s chi-square test. Logistic regression will be used for multivariable modeling with the same main effects as in aim 2 (symptom control, above). Final estimates will be reported as covariate-adjusted odds ratios.
- Transition to nursing home care: This outcome will be defined by whether patients transition to nursing home care during the time from discharge to 60 days follow-up. The proportions of those who do and do not transition to nursing home care will be compared using Pearson’s chi-square test. Logistic regression will be used for multivariable modeling with the same main effects as in aim 1. Final estimates will be reported as covariate-adjusted odds ratios.

#### Aim 3 analysis

The control and intervention arms will be compared to caregiver secondary outcomes at 60 days post-discharge.

- For the dichotomous outcome variables pertaining to communication about prognosis and goals of care and shared decision-making about hospitalization and shared decision-making about burdensome treatments, Pearson’s chi-square test will be used to test for differences in proportions between the study arms. We will use logistic regression for the multivariable modeling. Treatment effect estimates will be reported as adjusted odds ratios.
- To compare caregiver outcomes between intervention and control arms for caregiver distress (family distress in advanced dementia) [52], Student’s *t*-test for the mean difference will be used since the outcome is continuous. Linear mixed models as described above will be used to test treatment effects for 30-day and 60-day outcomes jointly.

### Interim analyses {21b}

No interim analyses are planned and this section is not applicable.

### Methods for additional analyses (e.g., subgroup analyses) {20b}

An exploratory analysis will be conducted to examine the interactions of the treatment group with study site, sex, race/ethnicity, and GDS stage on 60-day hospital transfers and on secondary outcomes.

### Methods in analysis to handle protocol non-adherence and any statistical methods to handle missing data {20c}

Missing data on independent variables are expected to be minimal. Simple mean and mode imputation for those variables will be included in regression models if less than 5% of patients have missing data; otherwise, conditional (i.e., regression) imputation will be used. Available case analysis will be conducted for 30-day outcomes. Missing 60-day dichotomous outcomes will be multiply-imputed if missingness exceeds 10%.

### Oversight and monitoring

#### Composition of the coordinating center and trial steering committee {5d}

The research team will be led by the PI and managed by the project manager reporting to the PI, who will provide administrative leadership and study coordination. This multi-site RCT will utilize a single IRB (UNC) and a unified protocol for all study sites. The UNC Cecil G. Sheps Health Services Research Center will be the administrative home for the UNC research investigators and staff. The project manager and data manager (UNC research staff) will facilitate data management, and data analysis will be led by the study biostatistician. The staff from the Palliative Care Research Cooperative Group’s Project Coordinating Center (University of Denver) will provide technical assistance to ensure good clinical practice, protocol version control, and central coordination of IRB tasks necessary for a large-scale multi-site clinical trial. Site-PIs in the research enrollment sites will oversee the study at UNC, IU, MGH/Harvard Medical School, UC, and Emory.

#### Composition of the data monitoring committee, its role, and reporting structure {21a}

The principal investigator (PI) will be responsible for ensuring the safety of participants daily. The Data and Safety Monitoring Board (DSMB), acting in an advisory capacity to the National Institute on Aging Director, will evaluate the progress of the study, review procedures for maintaining the confidentiality of data, the quality of data collection, management, and analyses.

#### Adverse event reporting and harms {22}

The occurrence of an adverse event (AE) or serious adverse event (SAE) may come to the attention of study personnel during study visits and interviews. Study-tracked AEs will include major emotional distress for family caregivers and confidentiality risk events for the person with ADRD. Deaths, life-threatening health events, or acute illness episodes will be tracked for all participants; however, these are expected health outcomes for people with late-stage ADRD and will not be included in AE reporting. All AEs, not otherwise precluded per the protocol, will be captured on the appropriate case report form (CRF) and classified for severity and potential relationship to the study procedures. All harms and AEs occurring while on study will be documented appropriately regardless of relationship. Potential AEs and SAEs will be collected systematically for all study participants at each study visit, when research team members will inquire about the occurrence of AE/SAEs since the last visit. Potential AEs and SAEs will be collected non-systematically (i.e., spontaneous reporting from open-ended questions), when the occurrence of an adverse event (AE) or serious adverse event (SAE) may come to the attention of study personnel when a study participant presents for medical care or upon review by a study monitor. Any medical or psychiatric condition that is present at the time that the participant is enrolled will be considered as a baseline and not reported as an AE.

Site-PIs will report any Unanticipated Problem Involving Risk to Subjects or Others (UPIRSO) or AEs to the PI and project manager in a timely manner. When a UPIRSO or AE is present, the PI and project manager will submit a report to the UNC IRB within 3 working days of receipt of this information. The reporting of any AE/SAE will be based on NIA and UNC IRB standards, on the severity of the AE (based on the Common Terminology Criteria for Adverse Events), its level of attribution to the intervention, and whether it is anticipated. All adverse events that are both serious (SAE) and unexpected would be reported to IRB, DSMB/SO, and NIA PO within 48 h of the study’s knowledge of SAE.

#### Frequency and plans for auditing trial conduct {23}

Summary of SAEs will be reported to NIA, PO, and DSMB/PO quarterly, unless otherwise requested by the DSMB. Summary of all AEs regardless of classification will be presented for each DSMB meeting.

#### Plans for communicating important protocol amendments to relevant parties (e.g., trial participants, ethical committees) {25}

All study protocols and amendments, informed consent forms, and other study materials will undergo review by the Institutional Review Board at UNC prior to initiating research and will be subject to annual and other required reviews. Any protocol changes will be reported to site-PIs. Changes will also be reported to study clinicians when these changes affect their roles.

#### Dissemination plans {31a}

This study will be conducted in accordance with the National Institutes of Health (NIH) Public Access Policy. It requires scientists to submit final peer-reviewed journal manuscripts that arise from NIH funds to the digital archive PubMed Central upon acceptance for publication. Second, this study will comply with the NIH Data Sharing Policy and Policy on the Dissemination of NIH-Funded Clinical Trial Information and the Clinical Trials Registration and Results Information Submission rule. As such, this trial will be registered at ClinicalTrials.gov, and results information from this trial will be submitted to ClinicalTrials.gov. In addition, every attempt will be made to publish results in peer-reviewed journals. Data from this study may be requested from other researchers 2 years after the completion of the primary endpoint by contacting the Palliative Care Research Collaborative Group [67]. Considerations for ensuring the confidentiality of these shared data are described above.

#### Biological specimens {33}

This section is not applicable.

## Discussion

The ADRD-PC study will be the first test of comprehensive dementia-specific palliative and transitional care. This study addresses the significant public health impact of late-stage ADRD and suffering due to symptom distress, burdensome treatments, and caregiver strain. While many caregivers prioritize comfort in late-stage ADRD, shared decision-making and access to specialty palliative care are rare. The ADRD-PC program leverages hospital palliative care teams to deliver comprehensive, dementia-specific palliative care plus transitional care. By including persons with GDS 5–7, ADRD-PC attends to the suffering of persons with ADRD and their caregivers along the illness trajectory rather than only at end-of-life.

The study protocol is designed with consideration for potential problems and challenges. Palliative care cannot be ethically withheld from patients in the control group, and investigators anticipate the potential for contamination. Routine palliative care consultation will be permitted for controls, but contamination risk is decreased since in current care this is very uncommon. Even when control patients are seen by palliative care specialists, these clinicians will lack ADRD-PC dementia-specific training and tools. Any palliative care consultation for control dyads will be tracked. Furthermore, as indicated in the ADRD-PC pilot, most attending physicians determined the need for palliative care consultation in the first 1–2 days of admission. Thus, these patients are more likely to be excluded than to create bias to the null. Second, while not found in the single-site pilot, barriers to physician referral may exist at new sites. Site-PIs will function as opinion leaders and (supported by the PI) will provide in-person education to overcome any resistance. Third, sites may have difficulty implementing the EHR screening algorithm. The study team will work with them on alternative screening and case-finding strategies compatible with local practice norms. Fourth, sites may find that lengths of stay are too short for some persons with ADRD; if this occurs, we will consider alternative strategies to deliver some elements of the ADRD intervention via virtual visits.

For persons with late-stage ADRD and their caregivers, there is an urgent need to improve outcomes of burdensome hospital transfers, symptom distress, shared decision-making, and caregiver distress. This study is responsive to the National Alzheimer’s Project Act, NIA priorities for geriatric palliative care, and quality care for ADRD. If efficacious, ADRD-PC has the potential for broad dissemination, as most US hospitals provide specialty palliative care.

## Trial status

The study protocol (NCT03810534), version number 1, was registered on July 2, 2021. Key trial implementation milestones were achieved. First, in all sites, ADRD-PC tools were added to electronic health record systems (EPIC) between July 23 and November 15, 2021. Second, in all sites, ADRD-PC intervention training was completed between June 14 and July 9, 2021. Third, in four study sites, dyad enrollment was started between July 27 and November 16, 2021.

Over the period from October to December 2021, the study team determined that COVID-related surges in hospital census were causing delays in participant enrollment. It was recognized that the original plan to achieve the sample within the study period was not feasible. Thus, in consultation with the NIA and the study DSMB, the study team developed a plan to add a fifth hospital to the multi-site RCT. The ADRD-PC protocol was revised, and the revised protocol (version 2.0) was approved by the UNC IRB on April 13, 2023, and updated on ClinicalTrials.gov (April 13, 2023). Subsequently, the ADRD-PC protocol was implemented in the fifth study site, Emory University (EU) Hospital in Atlanta, Georgia. In the EU Hospital, EPIC modifications and staff training were completed between March 7 and May 31, 2023, and enrollment of study participants was started on July 24, 2023. With the implementation of the ADRD-PC protocol in five hospitals, all participant enrollment is expected to be complete in May 2024.

Finally, the ADRD-PC protocol was updated to incorporate a secondary study of a Spanish version of the ADRD-PC intervention. The Spanish language ADRD-PC protocol will permit enrollment of up to 50 additional dyads, generating data to be used in separate analysis to describe the feasibility and acceptability of the Spanish version of ADRD-PC. The revised protocol was approved by the UNC IRB on April 13, 2023, and updated on ClinicalTrials.gov (April 13, 2023).

### Supplementary Information

**Additional file 1.** Supplementary Material.

**Additional file 2.**

## Data Availability

Reasonable requests will be considered by the authors with additional permission from the Institutional Review Board at the University of North Carolina at Chapel Hill.

## Abbreviations

ADRD: Alzheimer’s disease and related dementias
GDS: Global Deterioration Scale
ADRD-PC: Alzheimer’s Disease and Related Dementias-Palliative Care program
POLST: Physician Orders for Life-Sustaining Treatment
RCT: Randomized and controlled trial
USA: United States of America
SPIRIT: Standard Protocol Items: Recommendations for Interventional Trials
CONSORT: Consolidated Standards of Reporting Trials
UNC: University of North Carolina at Chapel Hill
MGH: Massachusetts General Hospital/Harvard Medical School
IU: Indiana University
UC: University of Colorado-Denver
LAR: Legally authorized representative
PI: Principal investigator
CRC: Clinical research coordinator
EHR: Electronic health record
NIH: National Institutes of Health
NIA: National Institute on Aging
SM-EOLD: Symptom Management – End of Life Dementia Questionnaire
NPI-Q: Neuropsychiatric Inventory Questionnaire
QUALID: Quality of Life in Late Dementia
BANS: Bedford Alzheimer Nursing Severity Scale
PHQ-2: Patient Health Questionnaire – 2
IRR: Incidence rate ratio
ID: Identification
CFR: Case report form
IRB: Institutional Review Board
DSMB: Data and Safety Monitoring Board
MZIP: Marginalized zero-inflated Poisson
AE: Adverse event
SAE: Serious adverse event
DSMB SO: Data and Safety Monitoring Board Safety Officer
NIA PO: National Institute on Aging Project Officer

## References

[1] Alzheimers Association. Alzheimer’s disease facts and figures. Chicago2023. Available from: https://www.alz.org/media/Documents/alzheimers-facts-and-figures.pdf.

[2] Harrison KL, Hunt LJ, Ritchie CS, Yaffe K (2019). Dying with dementia: underrecognized and stigmatized. J Am Geriatr Soc.

[3] Harrison KL, Ritchie CS, Patel K, Hunt LJ, Covinsky KE, Yaffe K (2019). Care settings and clinical characteristics of older adults with moderately severe dementia. J Am Geriatr Soc.

[4] Brodaty H, Breteler MM, Dekosky ST, Dorenlot P, Fratiglioni L, Hock C (2011). The world of dementia beyond 2020. J Am Geriatr Soc.

[5] Reisberg B, Ferris SH, de Leon MJ, Crook T (1982). The Global Deterioration Scale for assessment of primary degenerative dementia. Am J Psychiatry.

[6] Black BS, Finucane T, Baker A, Loreck D, Blass D, Fogarty L (2006). Health problems and correlates of pain in nursing home residents with advanced dementia. Alzheimer Dis Assoc Disord.

[7] Hanson LC, Eckert JK, Dobbs D, Williams CS, Caprio AJ, Sloane PD (2008). Symptom experience of dying long-term care residents. J Am Geriatr Soc.

[8] Hendriks SA, Smalbrugge M, Hertogh CM, van der Steen JT (2014). Dying with dementia: symptoms, treatment, and quality of life in the last week of life. J Pain Symptom Manage.

[9] Lyketsos CG, Carrillo MC, Ryan JM, Khachaturian AS, Trzepacz P, Amatniek J (2011). Neuropsychiatric symptoms in Alzheimer’s disease. Alzheimer’s Dement.

[10] Tosato M, Lukas A, van der Roest HG, Danese P, Antocicco M, Finne-Soveri H (2012). Association of pain with behavioral and psychiatric symptoms among nursing home residents with cognitive impairment: results from the SHELTER study. Pain.

[11] Jennings LA, Reuben DB, Evertson LC, Serrano KS, Ercoli L, Grill J (2015). Unmet needs of caregivers of individuals referred to a dementia care program. J Am Geriatr Soc.

[12] Vick JB, Ornstein KA, Szanton SL, Dy SM, Wolff JL (2019). Does caregiving strain increase as patients with and without dementia approach the end of life?. J Pain Symptom Manage.

[13] Gill TM, Gahbauer EA, Han L, Allore HG (2010). Trajectories of disability in the last year of life. N Engl J Med.

[14] Givens JL, Lopez RP, Mazor KM, Mitchell SL (2012). Sources of stress for family members of nursing home residents with advanced dementia. Alzheimer Dis Assoc Disord.

[15] Gozalo P, Teno JM, Mitchell SL, Skinner J, Bynum J, Tyler D (2011). End-of-life transitions among nursing home residents with cognitive issues. N Engl J Med.

[16] Teno JM, Gozalo PL, Bynum JP, Leland NE, Miller SC, Morden NE (2013). Change in end-of-life care for Medicare beneficiaries: site of death, place of care, and health care transitions in 2000, 2005, and 2009. JAMA.

[17] Fulton AT, Gozalo P, Mitchell SL, Mor V, Teno JM (2014). Intensive care utilization among nursing home residents with advanced cognitive and severe functional impairment. J Palliat Med.

[18] Teno JM, Mitchell SL, Skinner J, Kuo S, Fisher E, Intrator O (2009). Churning: the association between health care transitions and feeding tube insertion for nursing home residents with advanced cognitive impairment. J Palliat Med.

[19] Temkin-Greener H, Cen X, Hasselberg MJ, Li Y (2019). Preventable hospitalizations among nursing home residents with dementia and behavioral health disorders. J Am Med Dir Assoc.

[20] Givens JL, Selby K, Goldfeld KS, Mitchell SL (2012). Hospital transfers of nursing home residents with advanced dementia. J Am Geriatr Soc.

[21] Ingber MJ, Feng Z, Khatutsky G, Wang JM, Bercaw LE, Zheng NT (2017). Initiative to reduce avoidable hospitalizations among nursing facility residents shows promising results. Health Aff (Millwood).

[22] Loeb M, Carusone SC, Goeree R, Walter SD, Brazil K, Krueger P (2006). Effect of a clinical pathway to reduce hospitalizations in nursing home residents with pneumonia: a randomized controlled trial. JAMA.

[23] Hanson LC, Zimmerman S, Song MK, Lin FC, Rosemond C, Carey TS (2017). Effect of the goals of care intervention for advanced dementia: a randomized clinical trial. JAMA Intern Med.

[24] Mitchell SL, Palmer JA, Volandes AE, Hanson LC, Habtemariam D, Shaffer ML (2017). Level of care preferences among nursing home residents with advanced dementia. J Pain Symptom Manage.

[25] Mitchell SL, Teno JM, Kiely DK, Shaffer ML, Jones RN, Prigerson HG (2009). The clinical course of advanced dementia. N Engl J Med.

[26] Ashton SE, Roe B, Jack B, McClelland B (2016). End of life care: the experiences of advance care planning amongst family caregivers of people with advanced dementia - a qualitative study. Dementia (London, England).

[27] Biola H, Sloane PD, Williams CS, Daaleman TP, Zimmerman S (2010). Preferences versus practice: life-sustaining treatments in last months of life in long-term care. J Am Med Dir Assoc.

[28] Mitchell SL, Kiely DK, Hamel MB (2004). Dying with advanced dementia in the nursing home. Arch Intern Med.

[29] Sloane PD, Zimmerman S, Williams CS, Hanson LC (2008). Dying with dementia in long-term care. Gerontologist.

[30] Givens JL, Kiely DK, Carey K, Mitchell SL (2009). Healthcare proxies of nursing home residents with advanced dementia: decisions they confront and their satisfaction with decision-making. J Am Geriatr Soc.

[31] Tjia J, Dharmawardene M, Givens JL (2018). Advance directives among nursing home residents with mild, moderate, and advanced dementia. J Palliat Med.

[32] Goldsmith B, Dietrich J, Du Q, Morrison RS (2008). Variability in access to hospital palliative care in the United States. J Palliat Med.

[33] Miller SC, Han B (2008). End-of-life care in U.S. nursing homes: nursing homes with special programs and trained staff for hospice or palliative/end-of-life care. J Palliat Med.

[34] Center to Advance Palliative Care. State-by-state report card on access to palliative care 2019 [Available from: https://www.capc.org/report-card/.

[35] Center to Advance Palliative Care. About palliative care 2019 [Available from: https://www.capc.org/about/palliative-care/.

[36] Gaertner J, Siemens W, Meerpohl JJ, Antes G, Meffert C, Xander C (2017). Effect of specialist palliative care services on quality of life in adults with advanced incurable illness in hospital, hospice, or community settings: systematic review and meta-analysis. BMJ.

[37] Haun MW, Estel S, Rücker G, Friederich HC, Villalobos M, Thomas M (2017). Early palliative care for adults with advanced cancer. Cochrane Database Syst Rev.

[38] Casarett D, Pickard A, Bailey FA, Ritchie C, Furman C, Rosenfeld K (2008). Do palliative consultations improve patient outcomes?. J Am Geriatr Soc.

[39] DiMartino LD, Weiner BJ, Hanson LC, Weinberger M, Birken SA, Reeder-Hayes K (2018). Inpatient palliative care consultation and 30-day readmissions in oncology. J Palliat Med.

[40] Meier DE (2011). Increased access to palliative care and hospice services: opportunities to improve value in health care. Milbank Q.

[41] Hanson LC, Usher B, Spragens L, Bernard S (2008). Clinical and economic impact of palliative care consultation. J Pain Symptom Manage.

[42] Fazio S, Pace D, Maslow K, Zimmerman S, Kallmyer B (2018). Alzheimer’s Association Dementia Care Practice Recommendations. Gerontologist.

[43] Catic AG, Berg AI, Moran JA, Knopp JR, Givens JL, Kiely DK (2013). Preliminary data from an advanced dementia consult service: integrating research, education, and clinical expertise. J Am Geriatr Soc.

[44] Ernecoff NC, Wessell KL, Gabriel S, Carey TS, Hanson LC (2018). A novel screening method to identify late-stage dementia patients for palliative care research and practice. J Pain Symptom Manage.

[45] Mitchell SL, Catic AG, Givens JL, Knopp J, Moran JA (2011). Advanced dementia: a guide for families.

[46] Donabedian A (2005). Evaluating the quality of medical care 1966. Milbank Q.

[47] Mitchell PH, Lang NM (2004). Framing the problem of measuring and improving healthcare quality: has the Quality Health Outcomes Model been useful?. Med Care.

[48] Hanson LC, Kistler CE, Lavin K, Gabriel SL, Ernecoff NC, Lin FC (2019). Triggered palliative care for late-stage dementia: a pilot randomized trial. J Pain Symptom Manage.

[49] POLST N. POLST Basics 2022 [Available from: https://polst.org/about/.

[50] Kiely DK, Shaffer ML, Mitchell SL (2012). Scales for the evaluation of end-of-life care in advanced dementia: sensitivity to change. Alzheimer Dis Assoc Disord.

[51] Kaufer DI, Cummings JL, Ketchel P, Smith V, MacMillan A, Shelley T (2000). Validation of the NPI-Q, a brief clinical form of the Neuropsychiatric Inventory. J Neuropsychiatry Clin Neurosci.

[52] Givens JL, Jones RN, Mazor KM, Prigerson HG, Mitchell SL (2015). Development and psychometric properties of the family distress in advanced dementia scale. J Am Med Dir Assoc.

[53] Chan AW, Tetzlaff JM, Altman DG, Laupacis A, Gøtzsche PC, Krle AJK (2015). SPIRIT 2013 Statement: defining standard protocol items for clinical trials. Rev Panam Salud Publica.

[54] Schulz KF, Altman DG, Moher D (2011). CONSORT 2010 statement: updated guidelines for reporting parallel group randomised trials. Int J Surg.

[55] Onken LS, Carroll KM, Shoham V, Cuthbert BN, Riddle M (2014). Reenvisioning clinical science: unifying the discipline to improve the public health. Clin Psychol Sci.

[56] Hanson LC, Bull J, Wessell K, Massie L, Bennett RE, Kutner JS (2014). Strategies to support recruitment of patients with life-limiting illness for research: the Palliative Care Research Cooperative Group. J Pain Symptom Manage.

[57] Harris PA, Taylor R, Minor BL, Elliott V, Fernandez M, O’Neal L (2019). The REDCap consortium: building an international community of software platform partners. J Biomed Inform.

[58] Hansen LO, Young RS, Hinami K, Leung A, Williams MV (2011). Interventions to reduce 30-day rehospitalization: a systematic review. Ann Intern Med.

[59] Alzheimer’s Association. Late-stage caregiving 2023 [Available from: https://www.alz.org/help-support/caregiving/stages-behaviors/late-stage.

[60] Bellg AJ, Borrelli B, Resnick B, Hecht J, Minicucci DS, Ory M (2004). Enhancing treatment fidelity in health behavior change studies: best practices and recommendations from the NIH Behavior Change Consortium. Health Psychol.

[61] Jencks SF, Williams MV, Coleman EA (2009). Rehospitalizations among patients in the Medicare fee-for-service program. N Engl J Med.

[62] Zarit SH, Todd PA, Zarit JM (1986). Subjective burden of husbands and wives as caregivers: a longitudinal study. Gerontologist.

[63] Charlson ME, Pompei P, Ales KL, MacKenzie CR (1987). A new method of classifying prognostic comorbidity in longitudinal studies: development and validation. J Chronic Dis.

[64] Volicer L, Hurley AC, Lathi DC, Kowall NW (1994). Measurement of severity in advanced Alzheimer’s disease. J Gerontol.

[65] Kroenke K, Spitzer RL, Williams JB (2003). The patient health questionnaire-2: validity of a two-item depression screener. Med Care..

[66] Long DL, Preisser JS, Herring AH, Golin CE (2014). A marginalized zero-inflated Poisson regression model with overall exposure effects. Stat Med.

[67] Palliative Care Research Cooperative Group. A community of discovery 2023 [Available from: https://palliativecareresearch.org/.

