# Correction: Palliative care for persons with late-stage Alzheimer’s and related dementias and their caregivers: protocol for a randomized clinical trial

**DOI:** 10.1186/s13063-024-08260-0

**Published:** 2024-06-21

**Authors:** M. Toles, C. Kistler, F. C. Lin, M. Lynch, K. Wessell, S. L. Mitchell, L. C. Hanson

**Affiliations:** 1 School of Nursing, University of North Carolina at Chapel Hill, Chapel Hill, NC USA; 2 Department of Family Medicine and Palliative Care Program, School of Medicine, University of North Carolina at Chapel Hill, Chapel Hill, NC USA; 3 Department of Biostatistics, Gillings School of Global Public Health, University of North Carolina at Chapel Hill, Chapel Hill, NC USA; 4 Cecil G. Sheps Center for Health Services Research, University of North Carolina at Chapel Hill, Chapel Hill, NC USA; 5 Hebrew SeniorLifeHinda and Arthur Marcus Institute for Aging Research, and Department of Medicine, Beth Israel Deaconess Medical Center, Harvard Medical School, Boston, USA; 6 Division of Geriatrics and Palliative Care Program, School of Medicine, University of North Carolina at Chapel Hill, Chapel Hill, NC USA

**Correction** **:** **Trials 24, 606 (2023)**

**https://doi.org/10.1186/s13063-023-07614-4**

Following the publication of the original article [1], we have been notified that the study site mentioned under “Methods: Study Setting” should read “Eskenazi Health affiliated with Indiana University (IU)” instead of “Indiana University Health University Hospital”, as shown below:

“The study setting will be five US medical centers with interdisciplinary palliative care teams: University of North Carolina at Chapel Hill Medical Center (UNC), Massachusetts General Hospital/Harvard Medical School (MGH), Eskenazi Health affiliated with Indiana University (IU), UCHealth University of Colorado Hospital (UC), and Emory University Healthcare System (EU).”

The original article has been corrected.
