# Supplementary material for: Palliative care for persons with late-stage Alzheimer’s and related dementias and their caregivers: protocol for a randomized clinical trial
Source: Trials. 2023 Sep 25;24:606. doi: 10.1186/s13063-023-07614-4 (PMC10518941; doi:10.1186/s13063-023-07614-4)
Supplement: Supplementary file 1 — Additional file 1. Supplementary Material. [file 13063_2023_7614_MOESM1_ESM.docx]

**University of North Carolina at Chapel Hill**
**Consent to Participate in a Research Study**
**Adult Participants**

**Consent Form Version Date:** 04/05/2023
**IRB Study #** 20-2764
**Title of Study**: Palliative Care for Persons with Late-stage Alzheimer’s and Related Dementias and their Caregivers: a Randomized Clinical Trial
**Principal Investigator**: Laura Hanson
**Principal Investigator Department**: Med-Geriatric Medicine
**Principal Investigator Phone number**: (919) 843-4096
**Principal Investigator Email Address**: [lhanson@med.unc.edu](mailto:lhanson@med.unc.edu)
**Funding Source and/or Sponsor:** National Institutes of Health (NIH)

**Study Contact Telephone Number**: (919) 843-8874 or 919-966-7503
**Study Contact Email**: [molly_lynch@unc.edu](mailto:molly_lynch@unc.edu) or [briell@unc.edu](mailto:briell@unc.edu)

**CONCISE SUMMARY**
This study evaluates the Alzheimer’s Disease and Related Dementias Palliative Care (ADRD-PC) program of care for people with Alzheimer’s disease or other conditions causing dementia, and their family members.

You will be asked to tell us about your experience in 3 interviews, which should take less than 30 minutes each. The first will happen when you agree to participate, and the others will be 1 and 2 months after your family member leaves the hospital. As part of the study, we will also review your family member’s medical chart.

You will receive a total of $75 in gift cards if you complete all three interviews.

We don’t anticipate risks involved with this study. However, some individuals may find some of the questions in the interview uncomfortable and can skip these questions.

You may benefit personally by learning more about dementia care, and this may also help the person with dementia get better care.

**What are some general things you should know about research studies?**
You are being asked to take part in a research study.  To join the study is voluntary.

You can choose not to participate. Or you can withdraw at any time, for any reason, without any consequences to you or your family member.

Research studies are designed to obtain new knowledge. This new information may help people in the future.   You may not receive any direct benefit from being in the research study. There also may be risks to being in research studies. If you are a patient with an illness, you do not have to be in the research study in order to receive health care.

Details about this study are discussed below.  It is important that you understand this information so that you can make an informed choice about being in this research study. 

You will be given a copy of this consent form.  You should ask the researchers named above, or staff members who may assist them, any questions you have about this study at any time.

**What is the purpose of this study?**
The purpose of this research study is to evaluate the Alzheimer’s Disease and Related Dementias Palliative Care (ADRD-PC) program of care for people with dementia and their family members. For patients, we will learn if ADRD-PC helps to reduce hospitalizations, improve symptoms and support. For family members like you, we will learn if ADRD-PC helps improve communication with doctors and other clinicians and improve support.

**How many people will take part in this study?**
You are being asked to be in the study because you are a family member caring for someone living with dementia.
Approximately 474 pairs of family members and patients will participate in 5 locations -- University of North Carolina at Chapel Hill, Indiana University, University of Colorado, Massachusetts General Hospital and Emory University.

**How long will your part in this study last?**
Your part in the research study will last for two months after the patient leaves the hospital. There are two follow-up phone interviews during this two-month window, each taking less than 30 minutes.

**What will happen if you take part in the study?**
If you decide to participate you will be assigned randomly, or by chance, like flipping a coin, to a study group. People in Group 1 will consult with a specialist in Palliative Care. They will talk about how to help with dementia symptoms, and key decisions about treatment. They will also check in by phone about support needs after hospitalization. People in Group 2 will receive educational materials written by experts and tailored to help caregivers of someone with dementia.

While your family member is in the hospital, we’ll ask you to participate in a short interview, either in-person or over the telephone. We will call you 1 and 2 months after your family member leaves the hospital for follow-up telephone interviews You also have the option to complete the questions in a secure email or using a mailed paper copy. The interview has questions about your family member’s experience living with dementia, and your thoughts, feelings, and experiences being a caregiver for someone living with dementia.

**What are the possible benefits from being in this study?**
Research is designed to benefit everyone by gaining new knowledge. You are helping contribute to new knowledge that may benefit people like you in the future. Your participation is important to help us improve palliative consults and educational materials. You may gain satisfaction from helping the research that may be useful to other families in the future. You may benefit personally by learning more about dementia care, and this may also help the person with dementia get better care.

**What are the possible risks or discomforts involved from being in this study?**
We do not think you will experience any discomfort or risk from the study. However, talking about a family member’s illness may be emotionally stressful. We will work hard to make this a positive experience for you. You can always refuse to answer a question, or pause or even stop the interview.

**If you choose not to be in the study, what other treatment options do you have?
You do not have to be in this research study in order to receive regular medical care.**

**What if we learn about new findings or information during the study?**
You will be given any new information gained during the study that might affect your willingness to continue participating. 

**How will information about you be protected?**
Research studies have a small risk for some loss of privacy. To help prevent the loss of privacy, your name and the name of the person with dementia will not be recorded on any study documents. We will keep your family member’s health information private. We replace your names with a research ID number to be used with your interview answers or any other data. All files will be stored in a secure password protected database, on a secure computer. Only trained members of the research team will have access to the data.

The only time we would share information we learn about you or the person with dementia is if we have an immediate concern for your safety or well-being. If that happens, we will notify a member of your healthcare team with your permission.

Participants will not be identified in any report or publication about this study. We are required by law to store data from research studies afterwards in order to ensure that the findings can be re-checked and verified. Once all of the study data are analyzed, the information will be stored in a secure database overseen by the Palliative Care Research Cooperative Group (PCRC).

Consistent with the PCRC Data Sharing Policy, data will be stored without information that personally identifies people who participated. This “de-identified” data will be transferred and stored on a server maintained by the PCRC at the University of Colorado (U of CO), which are maintained and monitored by U of CO’s Office of Information Technology (OIT) Department to comply with good data storage and security practices.

All de-identified data that are collected and entered will remain in the PCRC Data Repositories indefinitely. This de-identified information could be used for future research studies or distributed to other investigators for future research studies without obtaining additional informed consent from you or your legally authorized representative. All future data analysis will be performed on de-identified data in the PCRC Data Repositories.  When information and data resulting from this study is presented at scientific meetings or published in a scientific journal, your identity cannot be revealed. Data will be presented in summary reports combining the information from many participants.

Every effort will be made to keep research records private. There may be times when federal or state law requires the disclosure of such records, including personal information.  This is very unlikely, but if disclosure is ever required, UNC-Chapel Hill will take steps allowable by law to protect the privacy of personal information.  In some cases, your information in this research study could be reviewed by representatives of the University, research sponsors, or government agencies (for example, the FDA) for purposes such as quality control or safety.

**What is a Certificate of Confidentiality?**
This research is covered by a Certificate of Confidentiality. With this Certificate, the researchers may not disclose or use information, documents or biospecimens that may identify you in any federal, state, or local civil, criminal, administrative, legislative, or other proceedings in the United States, for example, if there is a court subpoena, unless you have consented for this use. The Certificate cannot be used to refuse a request for information from personnel of a federal or state agency that is sponsoring the study for auditing or evaluation purposes or for information that must be disclosed in order to meet the requirements of the federal Food and Drug Administration (FDA).

The Certificate of Confidentiality will not be used to prevent disclosure as required by federal, state, or local law, such as mandatory reporting requirements for child abuse or neglect, disabled adult abuse or neglect, communicable diseases, injuries caused by suspected criminal violence, cancer diagnosis or benign brain or central nervous system tumors or other mandatory reporting requirement under applicable law. The Certificate of Confidentiality will not be used if disclosure is for other scientific research, as allowed by federal regulations protecting research subjects or for any purpose you have consented to in this informed consent document.

You should understand that a Certificate of Confidentiality does not prevent you from voluntarily releasing information about yourself or your involvement in this research. If an insurer, employer, or other person obtains your written consent to receive research information, then the researchers may not use the Certificate to withhold that information.

**What if you want to stop before your part in the study is complete?**
You can withdraw from this study at any time, without penalty.  The investigators also have the right to stop your participation at any time. This could be because you have had an unexpected reaction, or have failed to follow instructions, or because the entire study has been stopped.

If you withdraw from this study all data collected up until the point of withdrawal will be retained. No additional information will be collected unless you provide additional permission for further data collection at the time of your withdrawal.

**Will you receive anything for being in this study?**
You will be receiving a $25 gift card for completing the first interview and the 30- and 60-day interviews to thank you for your participation. You will receive a total of $75 in gift cards if you complete all three interviews. Any payment provided for participation in this study may be subject to applicable tax withholding obligations.

**Will it cost you anything to be in this study?**
Taking part of in this research study may lead to added costs for the patient’s insurance company for palliative care visits. These visits are considered standard care, and the patient’s insurance company may be charged for this care. The patient may be responsible for co-payments that are standard for their insurance.

**Who is sponsoring this study?**
This research is funded by National Institute of Health/National Institute on Aging.  This means that the research team is being paid by the sponsor for doing the study.  The researchers do not have a direct financial interest with the sponsor or in the final results of the study.

**What if you have questions about this study?**
You have the right to ask, and have answered, any questions you may have about this research. If you have questions about the study (including payments), complaints, concerns, or if a research-related injury occurs, you should contact the researchers listed on the first page of this form.

A description of this clinical trial will be available on www.clinicaltrials.gov, as required by U.S. Law. This website will not include information that can identify you. At most, the website will include a summary of the results. You can search this website at any time.

**What if you have questions about your rights as a research participant?**
All research on human volunteers is reviewed by a committee that works to protect your rights and welfare.  If you have questions or concerns about your rights as a research subject, or if you would like to obtain information or offer input, you may contact the Institutional Review Board at 919-966-3113 or by email to IRB_subjects@unc.edu.

| **Participant’s Agreement**:  I have read the information provided above.  I have asked all the questions I have at this time.   - Accepts participation in the study. - Declines participation in the study. - Is ineligible for the study. - Is unsure about participation in the study (🡪 arrange for additional contact with patient) |  |
| --- | --- |
|  |  |

| ______________________________________________________ Signature of Research Team Member Obtaining Consent | ____________________ Date of Verbal Consent |
| --- | --- |
| ______________________________________________________ Printed Name of Research Team Member Obtaining Consent |  |
